# Expression of the human antimicrobial peptide β-defensin-1 is repressed by the EGFR-ERK-MYC axis in colonic epithelial cells

**DOI:** 10.1038/s41598-018-36387-z

**Published:** 2018-12-21

**Authors:** Clément Bonamy, Emmanuel Sechet, Aurélien Amiot, Antoine Alam, Michael Mourez, Laurent Fraisse, Philippe J. Sansonetti, Brice Sperandio

**Affiliations:** 1 Unité de Pathogénie Microbienne Moléculaire, Institut Pasteur, 75015 Paris, France; 2 Unité INSERM U1202, Institut Pasteur, 75015 Paris, France; 3 Département de Gastroentérologie, Hôpital Henri Mondor, AP-HP, 94000 Créteil, France; 4 Sanofi, Infectious Diseases Therapeutic Area, 69280 Marcy l’Etoile, France; 5 Chaire de Microbiologie et Maladies Infectieuses, Collège de France, 75005 Paris, France

## Abstract

The human β-defensin-1 (HBD1) is an antimicrobial peptide constitutively expressed by epithelial cells at mucosal surfaces. In addition to its microbicidal properties, the loss of HBD1 expression in several cancers suggests that it may also have an anti-tumor activity. Here, we investigated the link between HBD1 expression and cancer signaling pathways in the human colon cancer cell lines TC7 and HT-29, and in normal human colonic primary cells, using a mini-gut organoid model. Using available datasets from patient cohorts, we found that HBD1 transcription is decreased in colorectal cancer. We demonstrated that inhibiting the Epidermal Growth Factor Receptor (EGFR) increased HBD1 expression, whereas activating EGFR repressed HBD1 expression, through the MEKK1/2-ERK1/2 pathway that ultimately regulates MYC. We finally present evidences supporting a role of MYC, together with the MIZ1 coregulator, in HBD1 regulation. Our work uncovers the role and deciphers the function of the EGFR-ERK-MYC axis as a repressor of HBD1 expression and contributes to the understanding of HBD1 suppression observed in colorectal cancer.

## Introduction

β-defensins are small cationic antimicrobial peptides from the innate immune response protecting mucosal surfaces against infections^1–3^. Among them, the human β-defensins-1 (HBD1) is constitutively and ubiquitously produced by epithelial cells, such as in the urinary tract, kidney tubules, pancreatic ducts, airways and intestine^4–6^. In addition, several hematopoietic cells, including dendritic cells and monocytes, express HBD1^7^. HBD1 action is predominantly directed against Gram-negative bacteria, the fungal *Candida* genus and enveloped viruses, such as HIV-1^8–10^.

Dysregulation of HBD1 gene transcription has been demonstrated in several types of cancers. Decreased expression of HBD1 was observed in both prostatic and renal carcinoma, suggesting its role as tumor suppressor in urological cancers^11–13^. A decrease in HBD1 expression was also found in oral squamous cell carcinoma, while HBD1 has been shown to suppress tumor migration and invasion and shown as a prognostic marker for oral squamous cell carcinoma^14–16^. Recently, HBD1 expression was found to be decreased in liver cancer and proposed to play a crucial role in liver cancer development^17^.

The Epidermal Growth Factor Receptor (EGFR) is a receptor tyrosine kinase commonly over-activated in cancers, such as glioblastoma (30–60%) and metastatic colorectal cancer (70–90%)^18–20^. Various mechanisms mediate the upregulation of EGFR activity, including mutations and truncations of its extracellular domain, as well as of its intracellular kinase domain^21^. These EGFR aberrations over-activate the downstream signaling pathways and transcription factors, including the MAPKs pathways and the MYC proto-oncogenic regulator^22^. In turn, these pathways activate or repress many biological functions that are beneficial to cancer cell proliferation.

The MYC transcription factor has a central role in cellular growth control, cell transformation and tumorigenesis^23^. At homeostasis, MYC expression is generally restricted to cells with regenerative and proliferative potential^24^. In contrast, MYC overexpression directly contributes to malignant transformation in various cell types and is a hallmark of many human cancers^25,26^. MYC is regulated both at the transcriptional and post-transcriptional levels and constitutes a direct target and effector of growth-regulatory cascades, like the EGFR pathway^27^. MYC heterodimerizes to bind the E-box DNA binding element CACGTG or variants thereof and to regulate, either positively or negatively, hundreds of genes^27,28^. Direct repression by MYC has been linked to its interaction with the MIZ1 coregulator^29,30^.

(i) Dysregulation of HBD1 expression in certain types of cancers, (ii) the putative activity of HBD1 as tumor suppressor, (iii) the relation between cancers and the EGFR pathway, and (iv) the presence of several putative E-box DNA binding sites for MYC in the HBD1 promoter prompted us to investigate the connection between regulation of HBD1 expression and cancer signaling pathways. We accordingly conducted an in-depth analysis to decipher the regulatory circuits influencing the constitutive expression of HBD1 in the human colon cancer cell lines TC7 and HT29, and in normal human colonic primary cells, using a mini-gut organoid model. Using publicly-available data sets of colorectal cancer patient, we showed that HBD1 is consistently downregulated in colon cancer compared to non-tumor colon specimens in 4 independent patient cohorts. We found that EGFR tyrosine kinase inhibitors and the monoclonal humanized anti-EGFR antibody Cetuximab, which are drugs approved for the treatment of several types of cancers, increased the constitutive expression of HBD1 *in vitro* and *ex vivo*. In contrast, we showed that the epidermal growth factor (EGF), the natural ligand of EGFR, had the opposite effect on HBD1 expression. We demonstrated that the MEKK1/2-ERK1/2 signaling cascade, which ultimately controls expression of the MYC transcription factor gene, mediates the EGFR-dependent regulation of HBD1. We finally provide molecular insights supporting a role of MYC in the EGFR-dependent control of HBD1 by binding its promoter, together with MIZ1, and impacting its transcription. Our work points out to the EGFR-MEKK1/2-ERK1/2-MYC axis which is dysregulated in many types of human cancers, as a regulatory circuit involved in the control of the HBD1 constitutive expression.

## Results

### Constitutive expression of HBD1 is decreased in colon cancer

**Figure 1:**
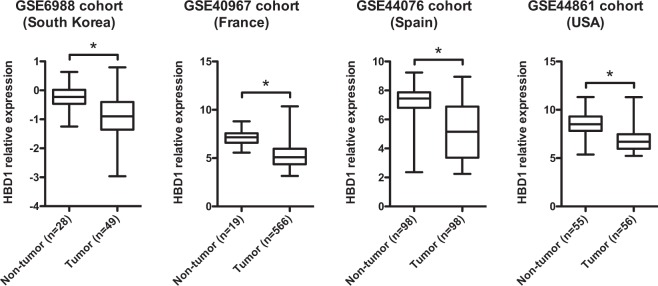
Constitutive expression of HBD1 is decreased in colon cancer. Transcription of the HBD1 gene in non-tumor and tumor specimens. Box plots are presented on a linear scale for the cohorts of patients GSE6988 (South Korea, n = 77), GSE40967 (France, n = 585), GSE44076 (Spain, n = 196) and GSE44861 (USA, n = 111). *P < 0,001 evaluated by Welch *t* test.

To investigate the transcription of β-defensin genes in tumors of patients with colorectal cancer, we extracted the probe sets for the β-defensin gene family available in the cohorts GSE6988 (South Korea)^31^, GSE40967 (France)^32^, GSE44076 (Spain)^33^ and GSE44861 (USA)^34^, with more than 77 specimens consisting of at least 49 samples of colon cancer in a single platform. β-defensin gene transcription was compared between non-tumor and tumor specimens. We found that HBD1 transcription was consistently decreased in colon cancer specimens, as compared to non-tumor specimens (Fig. 1). The decreased transcription of HBD1 in colon cancer specimens was statistically significant in the 4 cohorts (p < 0,001). In contrast, when data were available, no differences were observed in transcription of the β-defensin genes HBD2, HBD3 and HBD4, between tumor and non-tumor specimens (Fig S1). These observations underscore a decrease of the HBD1 constitutive expression in colon cancers, as previously reported for patients with prostatic, renal or liver cancers^11–13,17^.

### EGFR inhibition increases the constitutive expression of HBD1 *in vitro* and *ex vivo*

**Figure 2:**
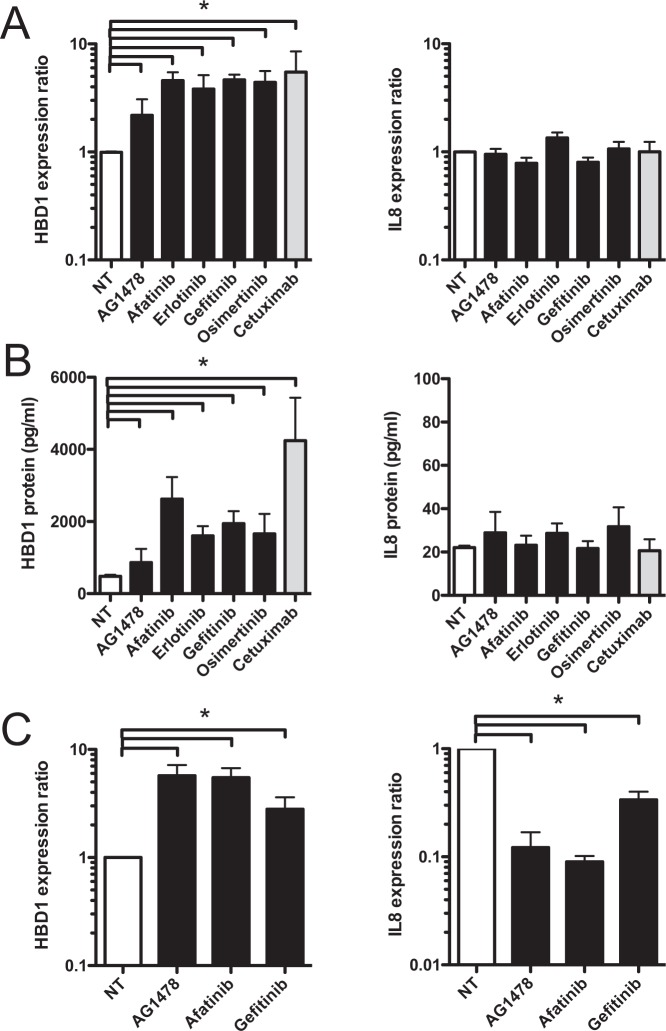
EGFR inhibition increases the constitutive expression of HBD1 *in vitro* and *ex vivo*. (**A**) Transcription of the HBD1 and IL8 genes in human colonic TC7 cells treated for 48 h with 1 μM of EGFR inhibitors AG1478, Afatinib, Erlotinib, Gefitinib and Osimertinib, or 100 nM Cetuximab. Values are presented on a logarithmic scale as the ratio of gene expression in treated cells compared with non-treated cells. NT, non-treated cells. *P < 0,05 evaluated by two-tailed Mann-Whitney *u* test. Data are represented as mean ± SD (n = 5 biological replicates). (**B**) ELISA dosage of the HBD1 and IL8 peptides secreted by TC7 cells treated for 48 h with 1 μM of EGFR inhibitors AG1478, Afatinib, Erlotinib, Gefitinib and Osimertinib, or 100 nM Cetuximab. Values are presented on a linear scale in picogram of peptide per milliliter. NT, non-treated cells. *P < 0,05 evaluated by two-tailed Mann-Whitney *u* test. Data are represented as mean ± SD (n = 5 biological replicates). (**C**) Transcription of the HBD1 and IL8 genes in human colonic organoids treated for 48 h with 1 μM AG1478, Afatinib, or Gefitinib. Values are presented on a logarithmic scale as the ratio of gene expression in treated organoids compared with non-treated organoids. NT, non-treated organoids. *P < 0,05 evaluated by two-tailed Mann-Whitney *u* test. Data are represented as mean ± SD (n = 5 biological replicates).

The previous observation that EGFR is overexpressed in around 50% of patients with colorectal cancers led us to investigate the implication of EGFR in the regulation of the constitutive expression of HBD1, by testing the effect of the FDA-approved EGFR tyrosine kinase inhibitors AG1478, Afatinib, Erlotinib, Gefitinib and Osimertinib, and the anti-EGFR humanized monoclonal antibody Cetuximab interacting with the EGFR extracellular binding site to block ligand stimulation^19,20^. Inhibitors and Cetuximab treatments at 1 μM and 100 nM, respectively, were performed *in vitro* on confluent monolayers of human colonic epithelial cells TC7^35^ and HT-29. RNA was extracted 48 h after treatment and analyzed by quantitative RT-PCR (qRT-PCR). In TC7 cells, treatment with the 5 inhibitors and Cetuximab increased the basic transcription of HBD1 from 2.5-fold (AG1478) to 5.5-fold (Cetuximab), as compared to non-treated cells (Fig. 2A). Similar results were obtained with HT-29 cells (Fig S2A). In contrast, transcription of the β-defensins HBD2 and HBD3, or the cytokines IL-1B, IL-8 and TNF used as markers of inflammation, was not modified by the inhibitors (Figs 2A and S2B).

To measure the secretion of the HBD1 peptide, ELISA dosages were performed on culture supernatants of TC7 cells treated for 48 h with EGFR inhibitors or Cetuximab. In the supernatants of non-treated cells, HBD1 was detected at its basal level of secretion, close to 450 pg/mL (Fig. 2B). Interestingly, supernatants of cells treated with inhibitors or Cetuximab contained increased concentrations of HBD1, up to 4200 pg/mL upon Cetuximab treatment. In contrast, the secretion of IL8 remained similar in treated and non-treated cells (Fig. 2B).

Then, we investigated the role of EGFR inhibition on transcription of HBD1 in human colonic primary cells, using *ex vivo* cultured organoids of colon tissues^36^. Crypts were isolated from normal human colons coming from several donor patients, and embedded in matrigel to induce the formation of organoids that recapitulate a tridimensional gut architecture harboring an internal lumen, stem cells located in surface protrusions corresponding to novel crypts, and the different epithelial lineages, including colonocytes. RNA was extracted from 5-day old organoids treated for 48 h with 1 μM AG1478, Afatinib, or Gefitinib and analyzed by qRT-PCR. As compared to non-treated organoids, transcription of HBD1 was increased from 2.8-fold (Gefitinib) to 5.8-fold (AG1478) in organoids treated with inhibitors (Fig. 2C). In contrast, transcription of IL8 was not increased upon exposure with inhibitors (Fig. 2C). Accordingly, inhibiting the EGFR pathway with EGFR tyrosine kinase inhibitors or the humanized antibody Cetuximab increased the constitutive expression of HBD1 *in vitro* and *ex vivo*.

### EGF decreases the constitutive expression of HBD1

**Figure 3:**
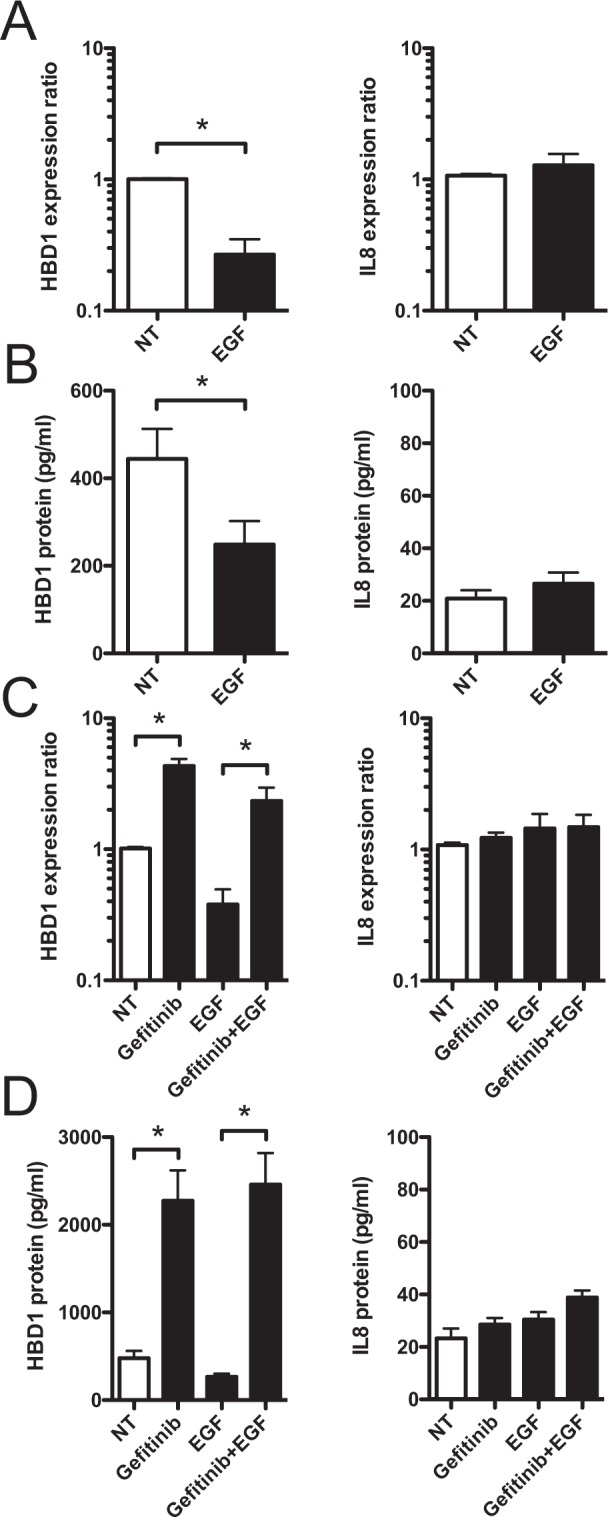
EGF decreases the constitutive expression of HBD1. (**A**) Transcription of the HBD1 and IL8 genes in TC7 cells treated for 48 h with 200 ng/mL EGF. Values are presented on a logarithmic scale as the ratio of gene expression in treated cells compared with non-treated cells. NT, non-treated cells. *P < 0,05 evaluated by two-tailed Mann-Whitney *u* test. Data are represented as mean ± SD (n = 7 biological replicates). (**B**) ELISA dosage of the HBD1 and IL8 peptides in supernatants of TC7 cells treated for 48 h with 200 ng/mL EGF. Values are presented on a linear scale in picogram of peptide per milliliter. NT, non-treated cells. *P < 0,05 evaluated by two-tailed Mann-Whitney *u* test. Data are represented as mean ± SD (n = 7 biological replicates). (**C**) Transcription of the HBD1 and IL8 genes in TC7 cells treated for 48 h with 1 μM Gefitinib, 200 ng/mL EGF, or 1 μM Gefitinib + 200 ng/mL EGF. Values are presented on a logarithmic scale as the ratio of gene expression in treated cells compared with non-treated cells. NT, non-treated cells. *P < 0,05 evaluated by two-tailed Mann-Whitney *u* test. Data are represented as mean ± SD (n = 4 biological replicates). (**D**) ELISA dosage of the HBD1 and IL8 peptides secreted by TC7 cells treated for 48 h with 1 μM Gefitinib, 200 ng/mL EGF, or 1 μM Gefitinib + 200 ng/mL EGF. Values are presented on a linear scale in picogram of peptide per milliliter. NT, non-treated cells. *P < 0,05 evaluated by two-tailed Mann-Whitney *u* test. Data are represented as mean ± SD (n = 4 biological replicates).

To analyze the impact of activation of the EGFR pathway by the EGF ligand on HBD1 expression, monolayers of TC7 and HT-29 cells were treated for 48 h with 200 ng/mL EGF and HBD1 transcription and secretion were analyzed by qRT-PCR and ELISA, respectively. In TC7 cells, EGF treatment induced a 4-fold decrease in HBD1 transcription and a 2-fold decrease in HBD1 secretion, as compared to non-treated cells (Fig. 3A,B). Similar results for HBD1 transcription were obtained with HT-29 cells (Fig S2A). As control, expression of IL8 was not modified in the same conditions (Fig. 3A,B).

We then analyzed the response of cells to treatment with Gefitinib in combination with EGF. Monolayers of confluent cells were treated for 48 h with 1 μM Gefitinib, 200 ng/mL EGF, or a combination of 1 μM Gefitinib and 200 ng/mL EGF. RNA and supernatants were collected and analyzed by qRT-PCR and ELISA, respectively (Fig. 3C,D). In cells treated with both Gefitinib and EGF, transcription and secretion of HBD1 were still increased as compared to EGF-treated cells, while IL8 expression remained unchanged. Together, these data demonstrated that activating the EGFR pathway by EGF decreases the constitutive expression of HBD1 and that blocking this pathway by the EGFR inhibitor Gefitinib counteracts this effect.

### The ERK signaling pathway mediates the EGFR-dependent repression of HBD1

**Figure 4:**
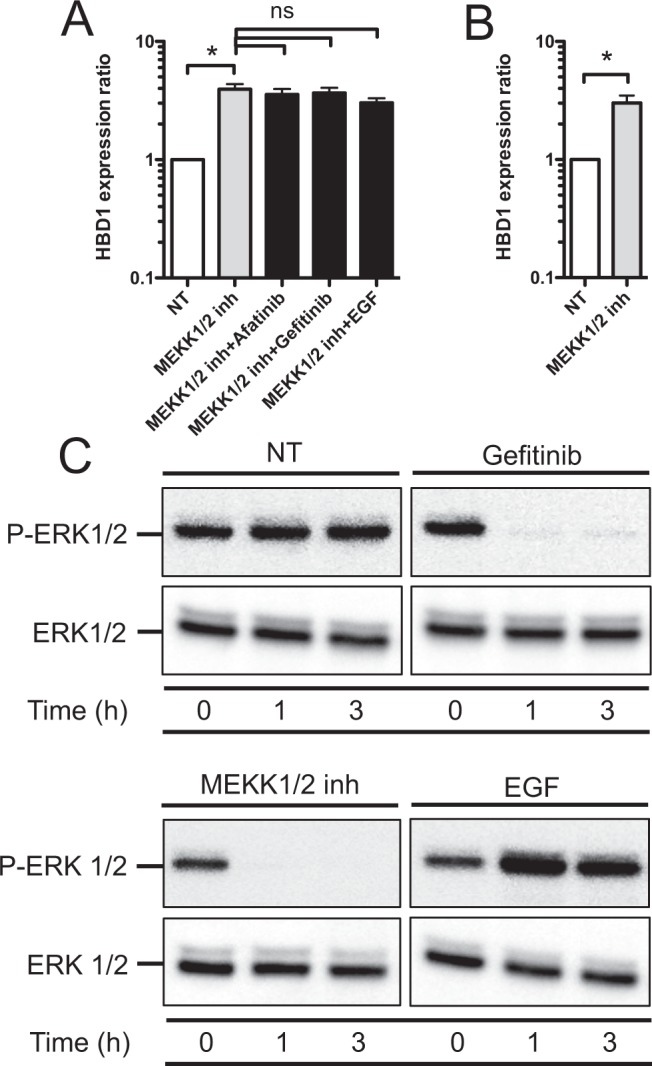
The ERK signaling pathway mediates the EGFR-dependent repression of HBD1. (**A**) Transcription of the HBD1 gene in TC7 cells treated for 48 h with 10 μM of the MEKK1/2 inhibitor (PD184352), 10 μM of the MEKK1/2 inhibitor + 1 μM Afatinib, 10 μM of the MEKK1/2 inhibitor + 1 μM Gefitinib, or 10 μM of the MEKK1/2 inhibitor + 200 ng/mL EGF. Values are presented on a logarithmic scale as the ratio of gene expression in treated cells compared with non-treated cells. NT, non-treated cells. *P < 0,05 evaluated by two-tailed Mann-Whitney *u* test; ns, not significant. Data are represented as mean ± SD (n = 5 biological replicates). (**B**) Transcription of the HBD1 gene in human colonic organoids treated for 48 h with 10 μM of the MEKK1/2 inhibitor (PD184352). Values are presented on a logarithmic scale as the ratio of gene expression in treated organoids compared with non-treated organoids. NT, non-treated organoids. *P < 0,05 evaluated by two-tailed Mann-Whitney *u* test. Data are represented as mean ± SD (n = 4 biological replicates). **(C)** Immunoblot analysis of ERK1/2 and phosphorylated ERK1/2 in TC7 cells treated or not with 1 μM Gefitinib, 10 μM of the MEKK1/2 inhibitor (PD184352), or 200 ng/mL EGF. After lysis of cells at the indicated time points, Western blots were performed using specific antibodies directed against proteins or phosphorylated proteins (representative of 3 biological replicates). “P” prefix, phosphorylation. NT, non-treated cells.

To determine which pathways downstream from EGFR activation transduced the signal modulating HBD1 transcription, we analyzed the potential involvement of the MEKK1/2, JNK, p38, PI3K and NF-κB cascades using the respectively relevant inhibitors PD184352, SP600125, SB203580, LY294002 and BMS345541. TC7 and HT29 cell monolayers and organoids were pretreated for 2 h with inhibitors and then treated or not for further 48 h with 1 μM Afatinib, 1 μM Gefitinib or 200 ng/mL EGF. Inhibiting MEKK1/2 increased HBD1 transcription in TC7 and HT-29 cells and organoids, whereas inhibiting JNK, p38, PI3K or NF-κB had the opposite effect (Figs 4A,B, S2A and S3A). HBD1 transcription in MEKK1/2-inhibited cells was not affected by treatment with Afatinib, Gefitinib or EGF (Figs 4A and S3B,C).

The phosphorylation status of MAPKs upon EGFR inhibition by Gefitinib, EGFR activation by EGF and MEKK1/2 inhibition by PD184352 was analyzed by immunoblot. We observed a strong decrease of ERK1/2 phosphorylation 1 h following treatment with Gefitinib and the MEKK1/2 inhibitor (Figs 4C and S6). In contrast, phosphorylation of ERK1/2 was increased upon EGF treatment (Figs 4C and S6), and phosphorylation of p38 and SAPK/JNK was not modified (Figs S4 and S7). These results show that the MEKK1/2-ERK1/2 signaling cascade is the main pathway involved in the EGFR-dependent regulation of HBD1 expression.

### The EGFR signaling pathway regulates MYC expression

**Figure 5:**
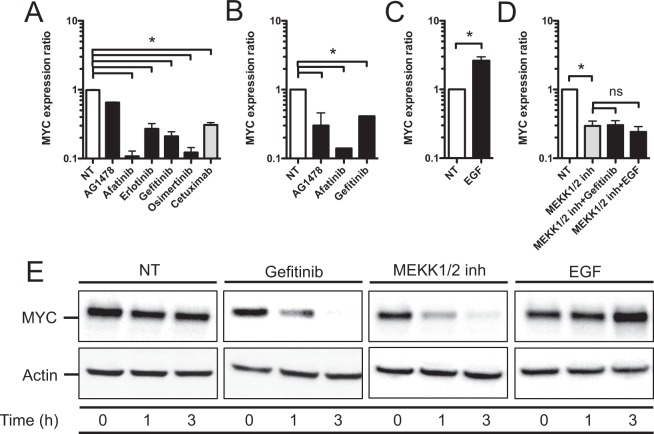
The EGFR signaling pathway regulates MYC expression. (**A**) Transcription of the MYC gene in TC7 cells treated for 48 h with 1 μM of the EGFR inhibitors AG1478, Afatinib, Erlotinib, Gefitinib and Osimertinib, or 100 nM Cetuximab. Values are presented on a logarithmic scale as the ratio of gene expression in treated cells compared with non-treated cells. NT, non-treated cells. *P < 0,05 evaluated by two-tailed Mann-Whitney *u* test. Data are represented as mean ± SD (n = 4 biological replicates). (**B**) Transcription of the MYC gene in human colonic organoids treated for 48 h with 1 μM AG1478, Afatinib, or Gefitinib. Values are presented on a logarithmic scale as the ratio of gene expression in treated organoids compared with non-treated organoids. NT, non-treated cells. *P < 0,05 evaluated by two-tailed Mann-Whitney *u* test. Data are represented as mean ± SD (n = 4 biological replicates). (**C**) Transcription of the MYC gene in TC7 cells treated for 48 h with 200 ng/mL EGF. Values are presented on a logarithmic scale as the ratio of gene expression in treated cells compared with non-treated cells. NT, non-treated cells. *P < 0,05 evaluated by two-tailed Mann-Whitney *u* test. Data are represented as mean ± SD (n = 7 biological replicates). (**D**) Transcription of the MYC gene in TC7 cells treated for 48 h with 10 μM of the MEKK1/2 inhibitor (PD184352), 10 μM of the MEKK1/2 inhibitor + 1 μM Gefitinib, or 10 μM of the MEKK1/2 inhibitor + 200 ng/mL EGF. Values are presented on a logarithmic scale as the ratio of gene expression in treated cells compared with non-treated cells. NT, non-treated cells. *P < 0,05 evaluated by two-tailed Mann-Whitney *u* test; ns, not significant. Data are represented as mean ± SD (n = 5 biological replicates). (**E**) Immunoblot analysis of MYC in TC7 cells treated or not with 1 μM Gefitinib, 10 μM of the MEKK1/2 inhibitor (PD184352), or 200 ng/mL EGF. After lysis of cells at the indicated time points, Western blots were performed using specific antibodies directed against proteins (representative of 3 biological replicates). NT, non-treated cells.

The transcription factor MYC is regulated both at the transcriptional and post-transcriptional levels and constitutes a direct target and an effector of growth-regulatory pathways^21,22^. To confirm the well-known regulation of MYC by the EGFR-MEKK1/2-ERK1/2 axis in our experimental models, we analyzed MYC transcription in TC7 cell monolayers treated for 48 h with EGFR tyrosine kinase inhibitors or Cetuximab. MYC transcription was decreased in cells treated with inhibitors or Cetuximab, compared to non-treated cells (Fig. 5A). This decrease in MYC transcription upon EGFR inhibition was confirmed *ex vivo* in organoids treated with EGFR tyrosine kinase inhibitors (Fig. 5B). In contrast, stimulating TC7 cells for 48 h with EGF led to a 2.8-fold increase in transcription of MYC (Fig. 5C). MYC transcription was also found to be increased in tumor specimens from the 4 cohorts of colorectal cancer patients (Fig S5). Inhibiting the MEKK1/2 pathway was led to a decrease in MYC transcription in cells treated or not with Gefitinib or EGF, validating that MEKK1/2 is the main pathway transducing the signal from EGFR to MYC (Fig. 5D).

We also analyzed MYC at the protein level upon EGFR inhibition with Gefitinib, EGFR activation with EGF, or MEKK1/2 inhibition with PD184352, by immunoblot. The amount of MYC was decreased as soon as 1 h following Gefitinib and MEKK1/2 inhibitor treatments, and slightly increased at 3 h upon EGF treatment (Figs 5E and S8). Together, these data confirmed that the EGFR-MEKK1/2-ERK1/2 axis controls MYC expression in our experimental models.

### MYC takes part to the EGFR-dependent repression of HBD1

**Figure 6:**
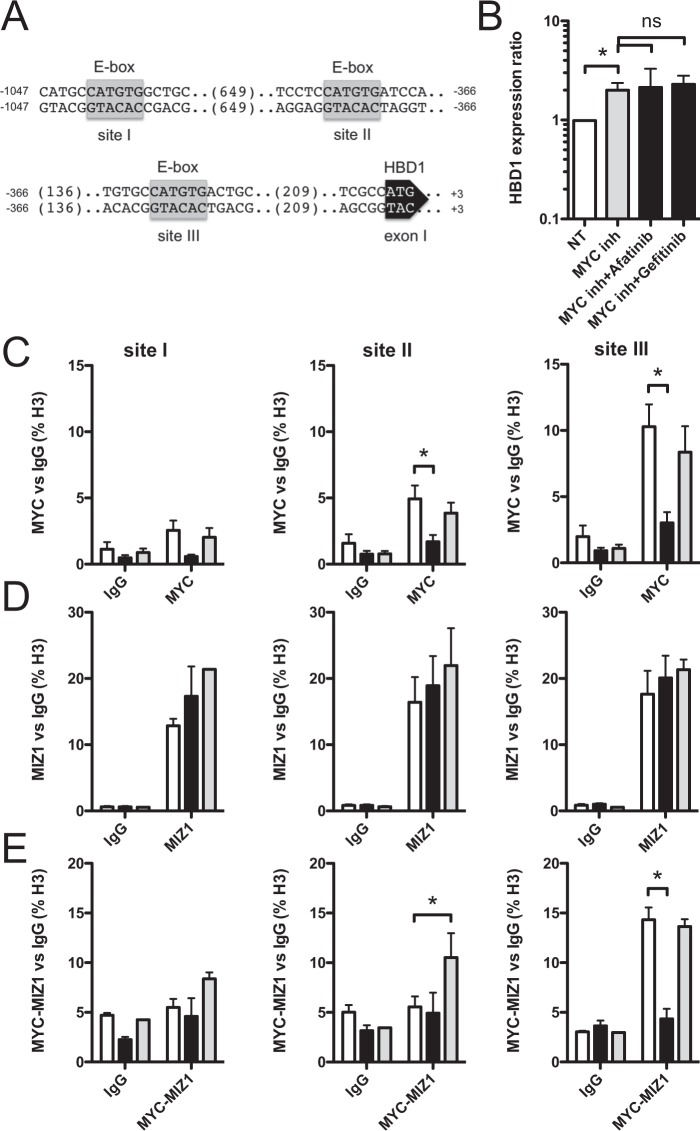
MYC mediates the EGFR-dependent repression of HBD1. (**A**) Promoter sequence of the human HBD1 gene. E-box DNA binding sites for MYC are indicated by grey boxes. (**B**) Transcription of the HBD1 gene in TC7 cells treated for 48 h with 500 nM of the MYC inhibitor (+)-JQ1, 500 nM of the MYC inhibitor (+)-JQ1 + 1 μM Afatinib, or 500 nM of the MYC inhibitor (+)-JQ1 + 1 μM Gefitinib. Values are presented on a logarithmic scale as the ratio of gene expression in treated cells compared with non-treated cells. NT, non-treated cells. *P < 0,05 evaluated by two-tailed Mann-Whitney *u* test; ns, not significant. Data are represented as mean ± SD (n = 5 biological replicates). (**C**–**E**) ChIP and ChIP-re-ChIP analysis of MYC (**C**), MIZ1 (**D**), and the MYC-MIZ1 complex (**E**) recruitment at the HBD1 promoter in cells treated with 1 μM Gefitinib (black bars), 200 ng/mL EGF (grey bars), or non-treated cells (white bars). Enrichment in chromatin was detected using MYC and/or MIZ1 specific antibodies, or rabbit IgG as control, and quantified by qRT-PCR using specific primers matching the HBD1 promoter at E-box sites (I, II, III). Values are presented as the percentage of signal relative to the histone H3 protein. *P < 0,05 evaluated by two-tailed Mann-Whitney *u* test. Data are represented as mean ± SD (n = 4 biological replicates).

Because (i) the EGFR pathway controls MYC and HBD1 transcription, and (ii) the promoter of HBD1 harbors three putative E-box DNA binding sites for MYC (Fig. 6A), we investigated the regulation of HBD1 by MYC. MYC functioning as a transcriptional activator or repressor depending on the targeted gene^37^, we tested the impact of its inhibition on HBD1 transcription using the inhibitor (+)-JQ1. TC7 cell monolayers were pretreated for 2 h with the inhibitor and then treated or not for further 48 h with 1 μM Afatinib or Gefitinib. Inhibiting MYC led to a 2-fold increase in HBD1 transcription, showing that MYC is a repressor of the HBD1 gene (Fig. 6B). HBD1 transcription in MYC-inhibited cells was similar with or without treatment with Afatinib and Gefitinib, indicating that MYC is one of the main transcription factors integrating the signal from the EGFR pathway to repress HBD1 transcription (Fig. 6B).

For many genes, repression by MYC relies on its ability to bind the targeted promoters with the coregulator MIZ1^29,30^. Therefore, we investigated the recruitment of MYC, MIZ1 and the MYC-MIZ1 complex at the HBD1 promoter upon activation and repression of the EGFR pathway 3 h after treatment with EGF and Gefitinib (Fig. 6C–E). Chromatin immuno-precipitation (ChIP) and ChIP-re-ChIP experiments were carried out using antibodies directed against the transcription factors or an irrelevant IgG of the same isotype as a control. Immunoprecipitated materials were analyzed by qRT-PCR, using the ribosomal protein RPL30 housekeeping gene as control. In non-treated cells, the signal corresponding to MYC recruitment increased from the E-boxes site I to site III, with ratios ranging from 2.5-fold to 10-fold (Fig. 6C), and the signal corresponding to MIZ1 recruitment was equivalent at the three E-boxes (Fig. 6D). In cells treated with EGF, signals were similar to those detected in non-treated cells for both proteins (Fig. 6C,D). In cells treated with Gefitinib, decreased recruitment of MYC was measured at the three E-boxes, as compared to non-treated cells (Fig. 6C), whereas MIZ1 recruitment was not impaired (Fig. 6D). These observations were confirmed by the study of the MYC-MIZ1 complex by ChIP-re-ChIP experiments (Fig. 6E). At the E-box site III, the MYC-MIZ1 complex was present in both non-treated and EGF-treated cells. In contrast, the complex was almost absent at the HBD1 promoter of Gefitinib-treated cells. Ratios decreased from 14-fold and 13-fold in non-treated cells and EGF-treated cells to 4-fold in Gefitinib-treated cells. These results showed that MYC and MIZ1 bind the HBD1 promoter to impact transcription of the HBD1 gene.

## Discussion

Little is known about the effectors from the human innate immune response that play a role in tumor suppression. It has been hypothesized that there is an imbalance between immune activation and suppression during cancer development, thus allowing tumor cells to escape immune recognition and destruction. As such, tumor progression has been linked to an increase in immune suppression in prostate cancer patients^38^. HBD1 has been proposed as a tumor suppressor given its properties to promote cancer cells apoptosis, cytolysis and its expression loss in tumor samples, such as prostate, kidney and liver cancers^12,13,17^. However, the molecular mechanism of the loss of HBD1 expression is unclear.

The presence of 5′ UTR single nucleotide polymorphisms in the HBD1 promoter was suggested to explain the loss or decrease of HBD1 expression^12^. Some cancer samples have been associated with the single nucleotide polymorphism C > G at -44 site (rs1800972) and correlate with lower HBD1 expression patterns. However, other studies contradict this hypothesis in that single nucleotide polymorphisms did not demonstrate a statistical difference in the decrease, suggesting that HBD1 loss may be related to other mechanisms^39–41^. Moreover, the single nucleotide polymorphisms hypothesis did not correlate with observations that loss of HBD1 expression occurred in tumor tissues, but was maintained in the adjacent normal tissues^11^.

Unlike a previous work claiming an activating role of MYC on HBD1^42^, here, we demonstrate that the EGFR-MEKK1/2-ERK1/2-MYC axis is involved in the regulation of HBD1 by repressing its expression in colonic epithelial cells. Blocking each step of this axis by the use of inhibitors targeting either EGFR (AG1478, Afatinib, Erlotinib, Gefitinib, Osimertinib, Cetuximab), MEKK1/2 (PD184352) or MYC (JQ-1), resulted in an increased expression of HBD1. It is well known that expression of the MYC transcription factor is aberrantly increased in several types of cancers, contributing to malignant transformation in various cell types^25^. Besides driving tumor initiation and progression, it is also well known that MYC is essential for tumor maintenance, malignant cells becoming addicted to continuous MYC overexpression^43,44^. We demonstrated that MYC impacts HBD1 transcription, by binding to the E-box DNA elements located within the HBD1 promoter. Furthermore, we showed that regulation of HBD1 by MYC occurs in association with the MIZ1 coregulator. Thus, given the putative tumor suppressor activity of HBD1, the delicate balance of the regulation of HBD1 gene expression exerted by MYC may play a key role in determining the fate of cancer cells and tumor progression.

In prostate cancer, the loss of HBD1 was shown to be due to a transcriptional repression exerted by the PAX2 oncogene^45^. Silencing of PAX2 expression in prostate cancer cells results in re-expression of HBD1. PAX2 is a transcriptional regulator normally expressed during early stages of development and is implicated as an oncogene in several types of cancers, where its expression is aberrantly increased^46–48^. It is known that PAX2 promotes cell survival through modification of expression of the p53 tumor suppressor. However, PAX2 negatively regulates cell death pathways in a p53-independent fashion^49^. The proposed mechanism supports the fact that PAX2 represses the activity of the HBD1 promoter by binding to a PAX2 DNA regulatory element located in the immediate vicinity of the HBD1 transcriptional start site^45^. Given (i) the fact that both PAX2 and MYC are repressors of HBD1 transcription and (ii) the proximity of their respective DNA recognition binding sites, it would be of interest to decipher their respective role in the suppression of HBD1 expression in colon cancer and whether they act in synergy to dampen HBD1 transcription.

The HBD1 gene has a mosaic promoter characterized by the presence of several DNA regulatory elements. In addition to MYC and PAX2, expression of HBD1 was shown to be also regulated by the NF-κB, HIF-1α and PPARγ transcription factors. NF-κB would mediate the upregulation of HBD1 observed upon proinflammatory situations, whereas HIF-1α and PPARγ would be critical for the maintenance of the HBD1 constitutive expression^50–54^. Beside HBD1 regulation, these transcription factors are also active players in human cancers. NF-κB plays a role in cancer initiation, development and metastasis, and a significant number of human cancers have constitutive NF-κB activity due to the inflammatory microenvironment and various oncogenic mutations^55^. HIF-1α targets the expression of several genes involved in many aspects of cancer biology including cell survival, glucose metabolism and cell invasion, and its over-expression has been associated with increased patient mortality in several cancer types^56^. PPARγ also plays a role in the regulation of cancer cell growth^57^. Thus, cancer development may affect HBD1 expression through several pathways and transcription factors that are normally, upon steady-state conditions, in charge to regulate the constitutive expression of HBD1. Deciphering their interconnection and prevailing activity would definitively help understanding the loss of HBD1 expression observed in colorectal cancer.

The capability of the immune system to recognize and kill cancer cells has become of great interest in cancer therapy researches. However, cancer cells have developed the ability to escape immune surveillance, despite evidences showing that immune effectors can play a crucial role in controlling tumor growth upon natural conditions or following treatments. Peptides from the innate immune response, like HBD1, can overcome these limitations through mechanisms of cancer cell destruction that involve membrane lysis and necrotic cell death. We are moving from the classical view of antimicrobial peptides as “anti-microbials” to a more complex view of their role in “anti-eukaryotic” immunity, anti-tumor especially. However, how these peptides achieve this role is not clear: (i) killing of emerging tumor cells in a normal epithelium by peptides produced by normal cells, (ii) regulation of the function of intratumor immune cells, (iii) both? The loss of expression of these peptides can be seen as another mechanism in which cancer cells evade destruction and promote tumorigenesis, therefore, approaches aiming to restore or boost expression of these peptides may represent a promising therapeutic strategy.

## Materials and Methods

### Microarray gene expression data extraction

Four datasets with more than 77 samples in a single microarray platform tested for gene expression in colorectal cancer were identified from the literature: GSE6988^31^, GSE40967^32^, GSE44076^33^, and GSE44861^34^. Data were extracted and analyzed as previously described^58^. Briefly, the GEO website has URLs for individual data sets (DataSeries as referred to on GEO) and for each GEO DataSeries, links are provided to the Series Matrix Files which contain the expression values for each gene (probeset) and each sample. Microarray gene expression data were retrieved from the data matrices deposited to GEO by the original authors. Data normalization methods were mentioned under each DataSeries on GEO. Once the gene expression matrices were successfully obtained, expression values were extracted for a small number of genes of interest, like HBD1 and MYC, and analyzed.

### Pharmacological inhibitors

Pharmacological inhibitors used in this work include: the EGFR inhibitors AG1478 (S2728, Selleckchem), Afatinib (S1011, Selleckchem; Giotrif), Erlotinib (S7786, Selleckchem; Tarceva), Gefitinib (S1025, Selleckchem; Iressa), Osimertinib (S7297, Selleckchem; Tagrisso), the MEKK1/2 inhibitor PD184352 (S1020, Selleckchem), the p38 inhibitor SB203580 (S1076, Selleckchem), the JNK inhibitor SP600125 (S1460, Selleckchem), the NF-κB inhibitors MG132 (S2619, Selleckchem) and BMS345541 (S8044, Selleckchem), the PI3K inhibitor LY294002 (S1105, Selleckchem), the MYC inhibitor (+)-JQ1 (S7110, Selleckchem).

### Antibodies

Antibodies used in this work include: Cetuximab (Creative Diagnostics Cat# AIT-19034, RRID:AB_2489605; Erbitux), anti-Erk1/2 (Cell Signaling Technology Cat# 4695, RRID:AB_390779), anti-phospho-ERK1/2 (T202/Y204) (Cell Signaling Technology Cat# 4370, RRID:AB_2315112), anti-p38 (Cell Signaling Technology Cat# 8690, RRID:AB_10999090), anti-phospho-p38 (T180/Y182) (Cell Signaling Technology Cat# 4511, RRID:AB_2139682), anti-SAPK/JNK (Cell Signaling Technology Cat# 9252, RRID:AB_2250373), anti-phospho-SAPK/JNK (T183/Y185) (Cell Signaling Technology Cat# 4668 P, RRID:AB_10831195), anti-MYC (Cell Signaling Technology Cat# 13987, RRID:AB_2631168), anti-MIZ1 (14300, Cell Signalling), anti-HBD1 (Abcam Cat# ab14425, RRID:AB_301206), anti-actin (Sigma-Aldrich Cat# A2066, RRID:AB_476693), anti-mouse IgG-POX (GE Healthcare Cat# NXA931-1ml, RRID:AB_772209), and anti-rabbit IgG-POX (GAR/IgG(H + L)/PO, Nordic Immunology).

### Cell culture

The human colonic epithelial cell lines Caco-2 (subclone TC7^35^) and HT-29 were cultured with DMEM (ThermoFisher) supplemented with 10% (vol/vol) decomplemented FBS (ThermoFisher), 1% nonessential amino acids (NEAA, ThermoFisher), 100 U/mL penicillin, and 100 μg/mL streptomycin (ThermoFisher) at 37 °C in 10% CO_2_. Cells were split two times per week using Versene solution (ThermoFisher). The TC7 cell line was a gift from Isabelle Chantret and Monique Rousset. This cell line is not listed in the database of commonly misidentified cell lines maintained by ICLAC. Cells were authenticated by microscopic observation and qRT-PCR experiment. Cells were tested negative for mycoplasma contamination.

### Human colonic organoid culture

This study was approved by the Institut Pasteur’s ethical and medical committee under the agreement N°2012-37. Surgically resected human colonic tissues were obtained from the Henri Mondor Hospital. All samples were obtained from patients who provided informed consent before surgery. Normal epithelia were isolated and cultured according to the protocol described by Sato *et al*., with minor modifications^36^. Organoids were cultured with Advanced DMEM/F12 (ThermoFisher) supplemented with Hepes (ThermoFisher), 2 mM GlutaMAX (ThermoFisher), 100 U/mL penicillin and 100 μg/mL streptomycin (ThermoFisher), 1× N2 and B27 supplements (ThermoFisher), 1 mM *N*-acetyl-L-cysteine (Sigma), 10 μM Y-27632 (Sigma), 500 nM A83-01 (Tocris), 10 μM SB202190 (Sigma), 10 mM nicotinamide (Sigma), 10 nM gastrin I (Sigma), 100 ng/mL recombinant human Noggin (R&D Systems), 50 ng/mL recombinant human EGF (R&D Systems), 1 μg/mL recombinant human R-Spondin-1 (Peprotech), 100 ng/mL recombinant human Wnt-3A (R&D Systems), 3 μM CHIR99021 (Sigma), and 10% (vol/vol) FBS (ThermoFisher), at 37 °C in 5% CO_2_. Oraganoids were cultured in 48-well plates, 100 crypts per well. RNA was isolated using the RNeasy Mini kit and the RNase free DNase kit (Qiagen). Gene expression was analyzed using primers purchased from Sigma. Data were normalized to the B2M housekeeping gene expression.

### qRT-PCR

RNA was isolated using the RNeasy Mini kit and the RNase free DNase kit (Qiagen). RT-PCR reactions were performed overnight using the SuperScript II reverse transcriptase (ThermoFisher) and the oligo(dT)18 primers (ThermoFisher), as recommended by the supplier. Gene-specific primers were designed and purchased from Sigma (HBD1, CAGGTGGTAACTTTCTCACAGG/AATAGAGACATTGCCCTCCACT; HBD2, GCCATGAGGGTCTTGTATCTC/TTAAGGCAGGTAACAGGATCG; HBD3, TTTGGTGCCTGTTCCAGGTCAT/GCCGCCTCTGACTCTGCAATAATA; IL1B, TACGATCACTGAACTGCACGCT/TCTTTCAACACGCAGGACAGGT; IL8, AAGAAACCACCGGAAGGAACCA/ATTTCTGTGTTGGCGCAGTGTG; TNF, AAACAACCCTCAGACGCCACAT/AGTGCTCATGGTGTCCTTTCCA; MYC, CAAACTTGAACAGCTACGGAAC/TTCATAGGTGATTGCTCAGGAC). The qRT-PCR reactions were carried out in a 20 μL final volume containing 8 μL of cDNA (diluted at 1/50), 2 μL of primers (0.2 μM each) and 10 μL of Power SYBR Green mix (ThermoFisher). Reactions were run on a QuantStudio 7 (ThermoFisher) with recommended universal thermal cycling parameters. Each sample reaction was run in duplicate on the same plate. Relative gene expression quantification was performed using the comparative Ct method. Data were normalized to the B2M housekeeping gene expression.

### ELISA

We used the ELISA kits for HBD1 (900-K202, PeproTech), and IL-8 (900-K18, PeproTech), as recommended by the supplier. Absorbance was measured on a M200PRO fluorimeter (Tecan).

### Immunoblotting

Total cell lysates were harvested by removing growth medium and adding Nonidet P-40 lysis buffer [25 mM Tris HCl (pH 7.5), 1 mM EDTA, 0.1 mM EGTA, 5 mM MgCl_2_, 1% Nonidet P-40, 10% (vol/vol) glycerol, 150 mM NaCl] supplemented by a mixture of protease inhibitors [sodium orthovanadate (Sigma), COMPLETE (Roche)]. Samples were diluted with sample buffer [1 M Tris HCl, 20% (vol/vol) glycerol, 6% (vol/vol) SDS, 0.02% bromophenol blue, 10% (vol/vol) β-mercaptoethanol] and boiled for 5 min. Denatured proteins were loaded on 7.5%, 10%, or 12% acrylamide Mini PROTEAN TGX precast gel (Bio-Rad). Separated proteins were transferred onto PVDF membrane using the Trans-Blot Turbo (Bio-Rad). Membranes were blocked with 3% (wt/vol) Albumin from Bovine Serum (BSA, Sigma) or 5% (wt/vol) milk (Regilait), at room temperature, before incubation with primary antibodies overnight at 4 °C, in 1% BSA or 5% (wt/vol) milk. Incubation with the secondary horseradish peroxidase-conjugated IgG antibody was performed at room temperature. Blots were developed using the SuperSignal West Dura Extended Duration Substrate solution (ThermoFisher) and the Amersham Imager 600 (GE). The presented results are representative of at least two independent experiments.

### Chromatin immuno-precipitation (ChIP) and ChIP-re-ChIP

ChIP was performed using the SimpleChIP Plus Enzymatic Chromatin IP Kit (Cell Signalling) using magnetic beads as recommended by the supplier. Chromatin inputs corresponded to 5–10 μg DNA for each individual ChIP assay. The ChIP DNA fractions were quantified by qRT-PCR on a QuantStudio 7 (ThermoFisher), using the comparative Ct method. Gene-specific primers were designed and purchased from Sigma (HBD1 site III GCGGCAGCCAGATGGAGACAAT/CCCTGGTGTCATTTGCCCTG; HBD1 site II CCTGCTCAGAGCTTCCCTGT/ACACTGGAGTCCCTCCTTCT; HBD1 site I CCACTCTGGGTGTCTCATGC/TCACGGTGGTCCAATGAGAA). For ChIP-re-ChIP experiments of the MYC-MIZ1 complex, chromatin inputs corresponded to 15–30 μg DNA per assay. The first immuno-precipitation was performed overnight using the anti-MIZ1 antibody. Beads were added and samples were incubated for 2 h at 4 °C on a wheel. After washing steps, chromatin was eluted and shared to perform the second immuno-precipitation using the anti-MYC antibody or controls (IgG and anti-H3 antibody).

### Statistics

Statistical analysis was performed on GraphPad Prism 5 (GraphPad software). Results are presented as a mean of at least 4 independent experiments. Error bars represent the SD. Statistical comparisons were performed using the two-tailed Mann-Whitney *u* test or the Welch *t* test. A p-value < 0.05 was considered significant. Pilot studies were performed and used for estimation of the sample size to ensure an adequate power. In most of our experiments, 4 to 6 samples were sufficient to see differences between experimental conditions. Samples were randomly assigned to experimental conditions, to processing order and to positions on plates where applicable. No blinding was done.

## Electronic supplementary material

Supplementary info

## Data Availability

The data that support the finding of this study are available from the corresponding author upon request.

## References

[1] Selsted ME, Ouellette AJ (2005). Mammalian defensins in the antimicrobial immune response. Nat. Immunol..

[2] Bevins CL, Salzman NH (2011). Paneth cells, antimicrobial peptides and maintenance of intestinal homeostasis. Nat. Rev. Microbiol..

[3] Gallo RL, Hooper LV (2012). Epithelial antimicrobial defence of the skin and intestine. Nat. Rev. Immunol..

[4] Zhao C, Wang I, Lehrer RI (1996). Widespread expression of beta-defensin hBD-1 in human secretory glands and epithelial cells. FEBS Lett..

[5] McCray PB, Bentley L (1997). Human airway epithelia express a beta-defensin. Am. J. Respir. Cell Mol. Biol..

[6] Tollin M (2003). Antimicrobial peptides in the first line defence of human colon mucosa. Peptides.

[7] Presicce P, Giannelli S, Taddeo A, Villa ML, Bella (2009). Della, S. Human defensins activate monocyte-derived dendritic cells, promote the production of proinflammatory cytokines, and up-regulate the surface expression of CD91. J. Leukoc. Biol..

[8] Schroeder BO (2011). Reduction of disulphide bonds unmasks potent antimicrobial activity of human β-defensin 1. Nature.

[9] Ricci E (2009). Role of beta-defensin-1 polymorphisms in mother-to-child transmission of HIV-1. J. Acquir. Immune Defic. Syndr..

[10] Alp S (2005). Expression of beta-defensin 1 and 2 in nasal epithelial cells and alveolar macrophages from HIV-infected patients. Eur. J. Med. Res..

[11] Donald CD (2003). Cancer-specific loss of beta-defensin 1 in renal and prostatic carcinomas. Lab. Invest..

[12] Sun CQ (2006). Human beta-defensin-1, a potential chromosome 8p tumor suppressor: control of transcription and induction of apoptosis in renal cell carcinoma. Cancer Res..

[13] Bullard RS (2008). Functional analysis of the host defense peptide Human Beta Defensin-1: new insight into its potential role in cancer. Mol. Immunol..

[14] Wenghoefer M (2008). Decreased gene expression of human beta-defensin-1 in the development of squamous cell carcinoma of the oral cavity. Int J Oral Maxillofac Surg.

[15] Joly S, Compton LM, Pujol C, Kurago ZB, Guthmiller JM (2009). Loss of human beta-defensin 1, 2, and 3 expression in oral squamous cell carcinoma. Oral Microbiol. Immunol..

[16] Han Q (2014). Human beta-defensin-1 suppresses tumor migration and invasion and is an independent predictor for survival of oral squamous cell carcinoma patients. Plos One.

[17] Ling Y-M (2017). β-defensin 1 expression in HCV infected liver/liver cancer: an important role in protecting HCV progression and liver cancer development. Sci Rep.

[18] Pines G, Köstler WJ, Yarden Y (2010). Oncogenic mutant forms of EGFR: lessons in signal transduction and targets for cancer therapy. FEBS Lett..

[19] Barber TD, Vogelstein B, Kinzler KW, Velculescu VE (2004). Somatic mutations of EGFR in colorectal cancers and glioblastomas. N. Engl. J. Med..

[20] Cappuzzo F (2008). EGFR FISH versus mutation: different tests, different end-points. Lung Cancer.

[21] Wee, P. & Wang, Z. Epidermal Growth Factor Receptor Cell Proliferation Signaling Pathways. *Cancers (Basel)* **9** (2017). 10.3390/cancers9050052 PMC5447962 28513565

[22] Meyer N, Penn LZ (2008). Reflecting on 25 years with MYC. Nat. Rev. Cancer.

[23] Marcu KB, Bossone SA, Patel AJ (1992). Myc function and regulation. Annu. Rev. Biochem..

[24] Wierstra I, Alves J (2008). The c-myc promoter: still MysterY and challenge. Adv. Cancer Res..

[25] Gabay, M., Li, Y. & Felsher, D. W. MYC activation is a hallmark of cancer initiation and maintenance. *Cold Spring Harb Perspect Med* **4** (2014). 10.1101/cshperspect.a014241 PMC4031954 24890832

[26] Ciriello G (2013). Emerging landscape of oncogenic signatures across human cancers. Nat. Genet..

[27] Eilers M, Eisenman RN (2008). Myc’s broad reach. Genes Dev..

[28] Blackwood EM, Eisenman RN (1991). Max: a helix-loop-helix zipper protein that forms a sequence-specific DNA-binding complex with Myc. Science.

[29] Herkert B, Eilers M (2010). Transcriptional repression: the dark side of myc. Genes Cancer.

[30] Schneider A, Peukert K, Eilers M, Hänel F (1997). Association of Myc with the zinc-finger protein Miz-1 defines a novel pathway for gene regulation by Myc. Curr. Top. Microbiol. Immunol..

[31] Ki DH (2007). Whole genome analysis for liver metastasis gene signatures in colorectal cancer. Int. J. Cancer.

[32] Marisa L (2013). Gene expression classification of colon cancer into molecular subtypes: characterization, validation, and prognostic value. PLoS Med..

[33] Cordero D (2014). Large differences in global transcriptional regulatory programs of normal and tumor colon cells. BMC Cancer.

[34] Ryan BM (2014). Germline variation in NCF4, an innate immunity gene, is associated with an increased risk of colorectal cancer. Int. J. Cancer.

[35] Chantret I (1994). Differential expression of sucrase-isomaltase in clones isolated from early and late passages of the cell line Caco-2: evidence for glucose-dependent negative regulation. J. Cell. Sci..

[36] Sato T (2011). Long-term expansion of epithelial organoids from human colon, adenoma, adenocarcinoma, and Barrett’s epithelium. Gastroenterology.

[37] Kress TR, Sabò A, Amati B (2015). MYC: connecting selective transcriptional control to global RNA production. Nat. Rev. Cancer.

[38] Palapattu GS (2005). Prostate carcinogenesis and inflammation: emerging insights. Carcinogenesis.

[39] Braida L (2004). A single-nucleotide polymorphism in the human beta-defensin 1 gene is associated with HIV-1 infection in Italian children. AIDS.

[40] Milanese M (2006). DEFB1 gene polymorphisms and increased risk of HIV-1 infection in Brazilian children. AIDS.

[41] Milanese, M., Segat, L. & Crovella, S. Transcriptional effect of DEFB1 gene 5′ untranslated region polymorphisms. *Cancer Res*. **67**, 5997–author reply 5997 (2007). 10.1158/0008-5472.CAN-06-3544 17575171

[42] Sherman H, Froy O (2008). Expression of human beta-defensin 1 is regulated via c-Myc and the biological clock. Mol. Immunol..

[43] Arvanitis C, Felsher DW (2006). Conditional transgenic models define how MYC initiates and maintains tumorigenesis. Semin. Cancer Biol..

[44] Felsher DW, Bishop JM (1999). Transient excess of MYC activity can elicit genomic instability and tumorigenesis. Proc. Natl. Acad. Sci. USA.

[45] Bose SK, Gibson W, Bullard RS, Donald CD (2009). PAX2 oncogene negatively regulates the expression of the host defense peptide human beta defensin-1 in prostate cancer. Mol. Immunol..

[46] Khoubehi B (2001). Expression of the developmental and oncogenic PAX2 gene in human prostate cancer. J. Urol..

[47] Muratovska A, Zhou C, He S, Goodyer P, Eccles MR (2003). Paired-Box genes are frequently expressed in cancer and often required for cancer cell survival. Oncogene.

[48] Silberstein GB, Dressler GR, Van Horn K (2002). Expression of the PAX2 oncogene in human breast cancer and its role in progesterone-dependent mammary growth. Oncogene.

[49] Gibson W, Green A, Bullard RS, Eaddy AC, Donald CD (2007). Inhibition of PAX2 expression results in alternate cell death pathways in prostate cancer cells differing in p53 status. Cancer Lett..

[50] O’Neil DA (1999). Expression and regulation of the human beta-defensins hBD-1 and hBD-2 in intestinal epithelium. J. Immunol..

[51] Prado-Montes de Oca E, Velarde-Félix JS, Ríos-Tostado JJ, Picos-Cárdenas VJ, Figuera LE (2009). SNP 668C (−44) alters a NF-kappaB1 putative binding site in non-coding strand of human beta-defensin 1 (DEFB1) and is associated with lepromatous leprosy. Infect. Genet. Evol..

[52] Kelly CJ (2013). Fundamental role for HIF-1α in constitutive expression of human β defensin-1. Mucosal Immunol.

[53] Peyrin-Biroulet L (2010). Peroxisome proliferator-activated receptor gamma activation is required for maintenance of innate antimicrobial immunity in the colon. Proc. Natl. Acad. Sci. USA.

[54] Martin H, McGhie TK, Bentley-Hewitt K, Christeller J (2013). PPARγ as a sensor of lipase activity and a target for the lipase inhibitor orlistat. Lipids Health Dis.

[55] Xia Y, Shen S, Verma IM (2014). NF-κB, an active player in human cancers. Cancer Immunol Res.

[56] Quintero M, Mackenzie N, Brennan PA (2004). Hypoxia-inducible factor 1 (HIF-1) in cancer. Eur J Surg Oncol.

[57] Tachibana K, Yamasaki D, Ishimoto K, Doi T (2008). The Role of PPARs in Cancer. PPAR Res.

[58] Kwok HF (2015). Prognostic significance of minichromosome maintenance proteins in breast cancer. Am J Cancer Res.

