# Supplementary material for: Expression of the human antimicrobial peptide β-defensin-1 is repressed by the EGFR-ERK-MYC axis in colonic epithelial cells
Source: Sci Rep. 2018 Dec 21;8:18043. doi: 10.1038/s41598-018-36387-z (PMC6303337; doi:10.1038/s41598-018-36387-z)
Supplement: Supplementary file 1 — Supplementary info [file 41598_2018_36387_MOESM1_ESM.docx]

**Supplementary information**

**Expression of the human antimicrobial peptide β-defensin-1 is repressed by the EGFR-ERK-MYC axis in colonic epithelial cells**

Clément Bonamy, Emmanuel Sechet, Aurélien Amiot, Antoine Alam, Michael Mourez, Laurent Fraisse, Philippe J. Sansonetti, and Brice Sperandio

**Figure S1: Transcription of the HBD2, HBD3 and HBD4 genes is not modified in colorectal cancer.** Transcription of the HBD2, HBD3 and HBD4 genes in non-tumor and tumor specimens. Box plots are presented on a logarithmic scale for the cohorts of patients **(A)** GSE40967 (France, n=586) and **(B)** GSE44076 (Spain, n=196). Data are not available for cohorts GSE6988 and GSE44861. P-value is evaluated by Welch *t* test.

**Figure S2: Transcription of antimicrobial peptide or proinflammatory genes upon EGFR inhibition in HT-29 or TC7 cells. (A)** Transcription of the HBD1 gene in human colonic HT-29 cells treated for 48 h with 1 μM of the EGFR inhibitor AG1478, 100 nM Cetuximab, 200 ng/mL EGF, or 10 μM of the MEKK1/2 inhibitor PD184352. Values are presented on a logarithmic scale as the ratio of gene expression in treated cells compared with non-treated cells. NT, non-treated cells. *P < 0,05 evaluated by two-tailed Mann-Whitney *u* test. Data are represented as mean ± SD (n=3 biological replicates). **(B)** Transcription of the HBD2, HBD3, IL1B and TNF genes in TC7 cells treated for 48 h with 1 μM Gefitinib. Values are presented on a logarithmic scale as the ratio of gene expression in treated cells compared with non-treated cells. Data are represented as mean ± SD (n=3 biological replicates).

**Figure S3: The EGFR-dependent regulation of HBD1 transcription is not mediated by the JNK, p38, PI3K, or NF-κB signaling pathways. (A)** Transcription of the HBD1 gene in TC7 cells treated for 48 h with 10 μM of the JNK inhibitor (SP600125), 10 μM of the p38 inhibitor (SB203580), 1 μM of the PI3K inhibitor (LY29002), or 10 μM of the NF-κB inhibitor (BMS-345541). Values are presented on a logarithmic scale as the ratio of gene expression in treated cells compared with non-treated cells. NT, non-treated cells. *P < 0,05 evaluated by two-tailed Mann-Whitney *u* test. Data are represented as mean ± SD (n=6 biological replicates). **(B)** Transcription of the HBD1 gene in TC7 cells treated for 48 h with 1 μM Gefitinib, 10 μM of the JNK inhibitor (SP600125), 10 μM of the JNK inhibitor (SP600125) + 1 μM Gefitinib, 10 μM of the p38 inhibitor (SB203580), 10 μM of the p38 inhibitor (SB203580) + 1 μM Gefitinib, 1 μM of the PI3K inhibitor (LY29002), 1 μM of the PI3K inhibitor (LY29002) + 1 μM Gefitinib, 10 μM of the NF-κB inhibitor (BMS-345541), or 10 μM of the NF-κB inhibitor (BMS-345541) + 1 μM Gefitinib. Values are presented on a logarithmic scale as the ratio of gene expression in treated cells compared with non-treated cells. NT, non-treated cells. *P < 0,05 evaluated by two-tailed Mann-Whitney *u* test. Data are represented as mean ± SD (n=3 biological replicates). **(C)** Transcription of the HBD1 gene in TC7 cells treated for 48 h with 200 ng/mL EGF, 10 μM of the JNK inhibitor (SP600125), 10 μM of the JNK inhibitor (SP600125) + 200 ng/mL EGF, 10 μM of the p38 inhibitor (SB203580), 10 μM of the p38 inhibitor (SB203580) + 200 ng/mL EGF, 1 μM of the PI3K inhibitor (LY29002), 1 μM of the PI3K inhibitor (LY29002) + 200 ng/mL EGF, 10 μM of the NF-κB inhibitor (BMS-345541), or 10 μM of the NF-κB inhibitor (BMS-345541) + 200 ng/mL EGF. Values are presented on a logarithmic scale as the ratio of gene expression in treated cells compared with non-treated cells. NT, non-treated cells. *P < 0,05 evaluated by two-tailed Mann-Whitney *u* test. Data are represented as mean ± SD (n=3 biological replicates).

**Figure S4: Phosphorylation status of p38 and JNK upon EGFR and MEKK1/2 inhibition, or EGFR activation.** Immunoblot analysis of p38, phosphorylated p38, SAPK/JNK and phosphorylated SAPK/JNK in TC7 cells treated or not with 1 μM Gefitinib, 10 μM of the MEKK1/2 inhibitor (PD184352), or 200 ng/mL EGF. After lysis of cells at the indicated time point, Western blots were performed using specific antibodies directed against proteins or phosphorylated proteins (representative of 3 biological replicates). “P” prefix, phosphorylation. NT, non-treated cells.

**Figure S5: Increased transcription of MYC in colorectal cancer.** Transcription of the MYC gene in non-tumor and tumor specimens. Box plots are presented on a linear scale for the cohorts of patients GSE6988 (South Korea, n=77), GSE40967 (France, n=586), GSE44076 (Spain, n=196), and GSE44861 (USA, n=111). *P < 0,001 evaluated by Welch *t* test.

**Figure S6: Full-length blots presented in Figure 4. (A, C)** Western-blots revealed with anti-phospho-ERK1/2 (T202/Y204) (Cell Signaling Technology Cat# 4370, RRID:AB_2315112). **(B, D)** Western-blots revealed with anti-ERK1/2 (Cell Signaling Technology Cat# 4695, RRID:AB_390779).

**Figure S7: Full-length blots presented in Figure S4. (A)** Western-blot revealed with anti-phospho-p38 (T180/Y182) (Cell Signaling Technology Cat# 4511, RRID:AB_2139682). **(B)** Western-blot revealed with anti-p38 (Cell Signaling Technology Cat# 8690, RRID:AB_10999090). **(C)** Western-blot revealed with anti-phospho-SAPK/JNK (T183/Y185) (Cell Signaling Technology Cat# 4668P, RRID:AB_10831195). **(D)** Western-blot revealed with anti-SAPK/JNK (Cell Signaling Technology Cat# 9252, RRID:AB_2250373).

**Figure S8: Full-length blots presented in Figure 5. (A, C)** Western-blots revealed with anti-MYC (Cell Signaling Technology Cat# 13987, RRID:AB_2631168). **(B, D)** Western-blots revealed with anti-actin (Sigma-Aldrich Cat# A2066, RRID:AB_476693).

**Figure S1**

**
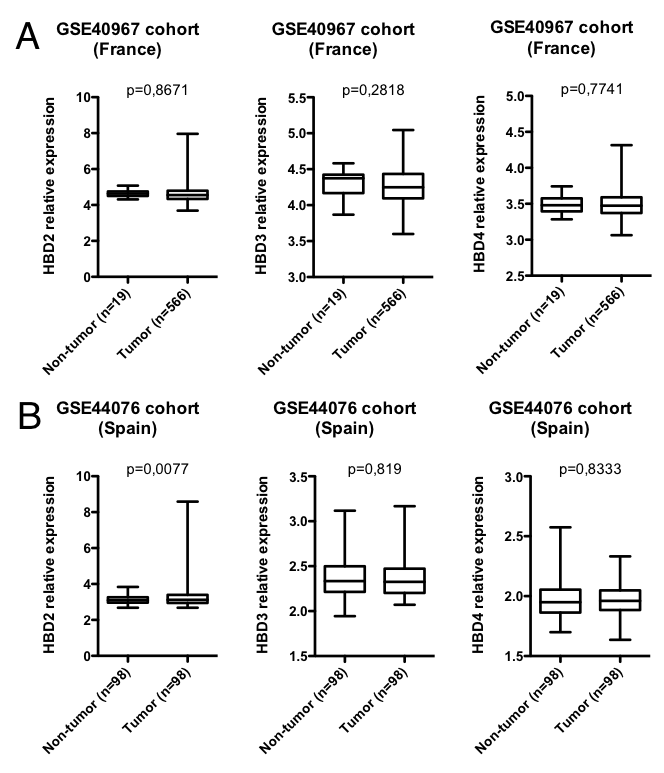
**

**Figure S2**

**
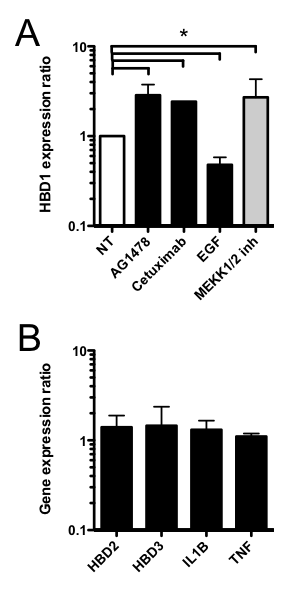
**

**Figure S3**

**
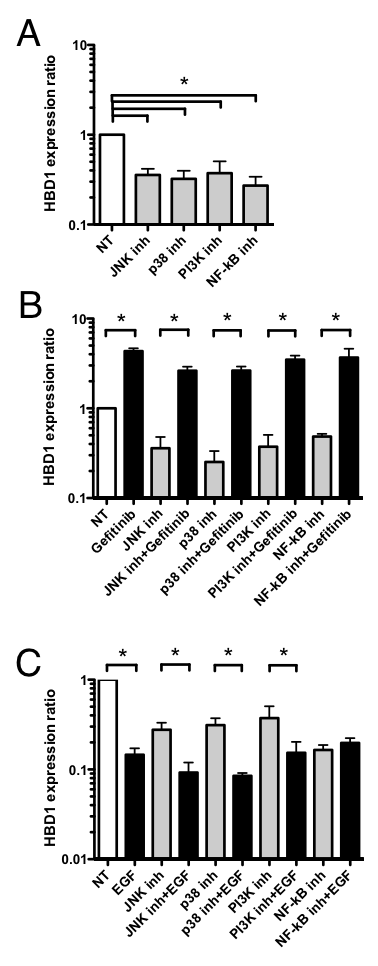
**

**Figure S4**

**
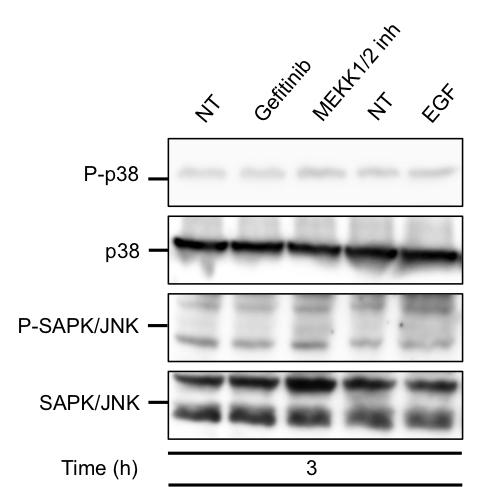
**

**Figure S5**

**
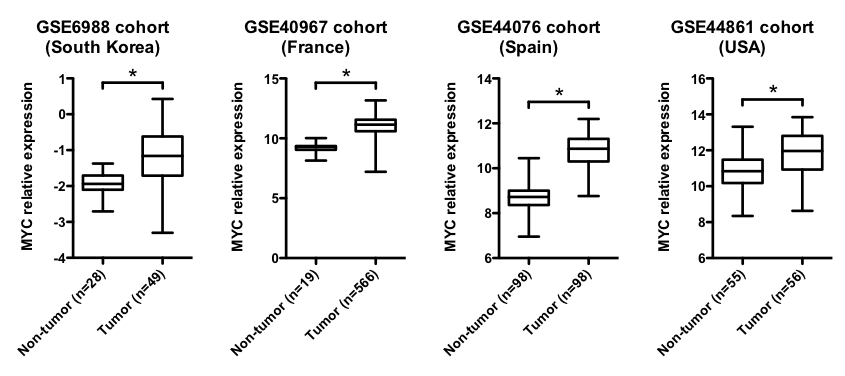
**

**Figure S6**

**
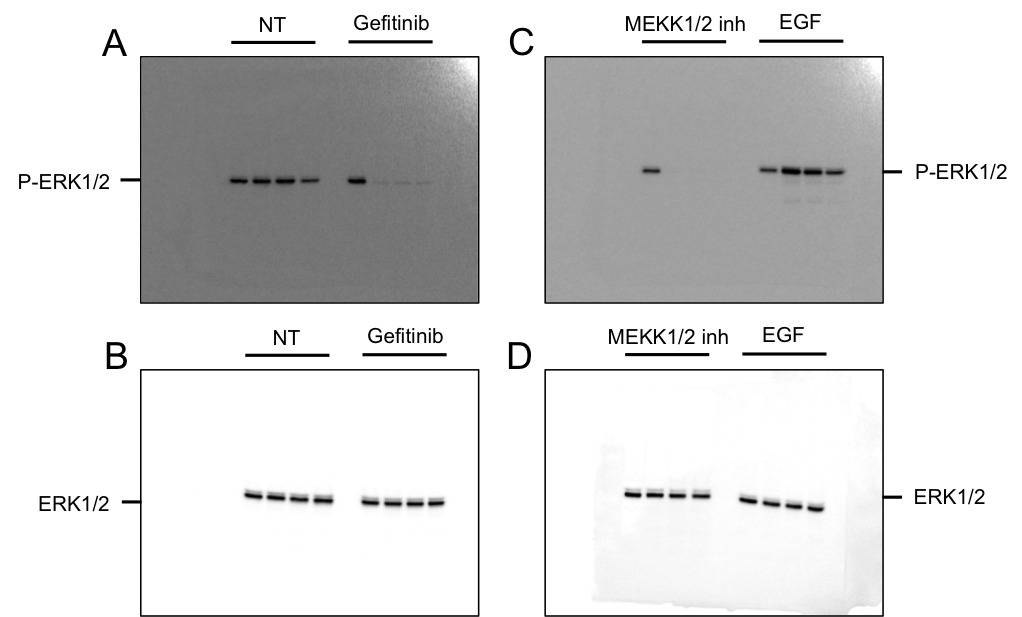
**

**Figure S7**

**
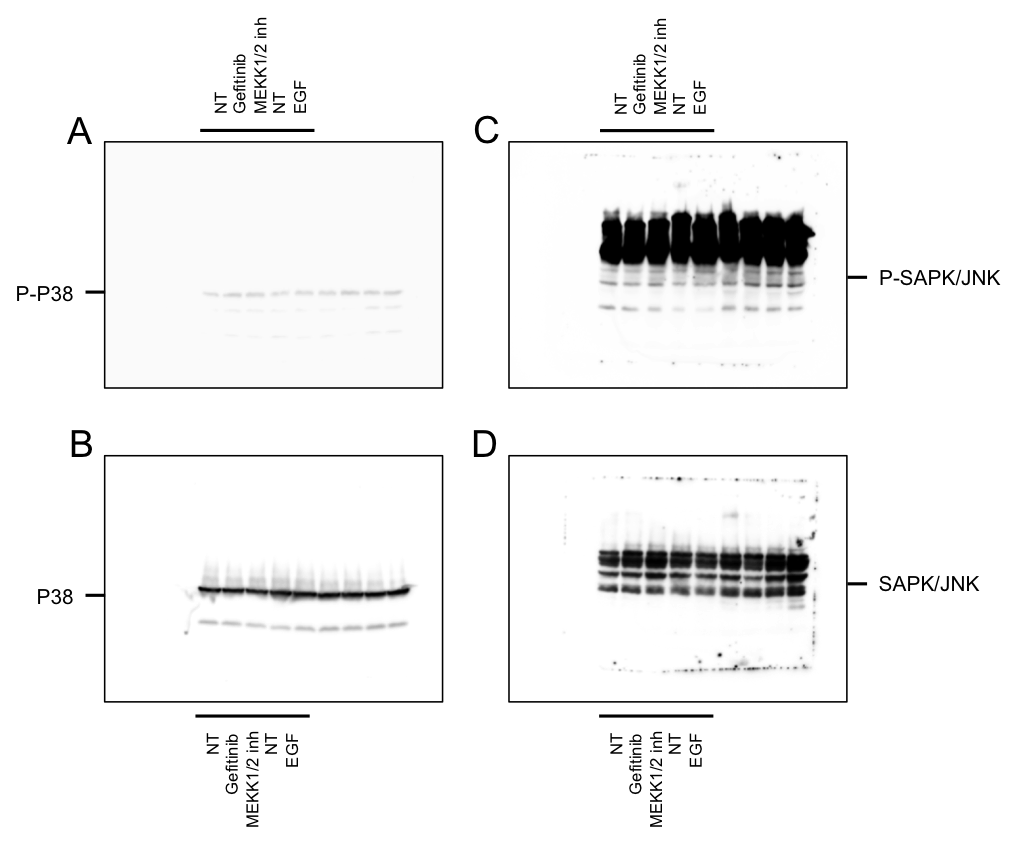
**

**Figure S8**

**
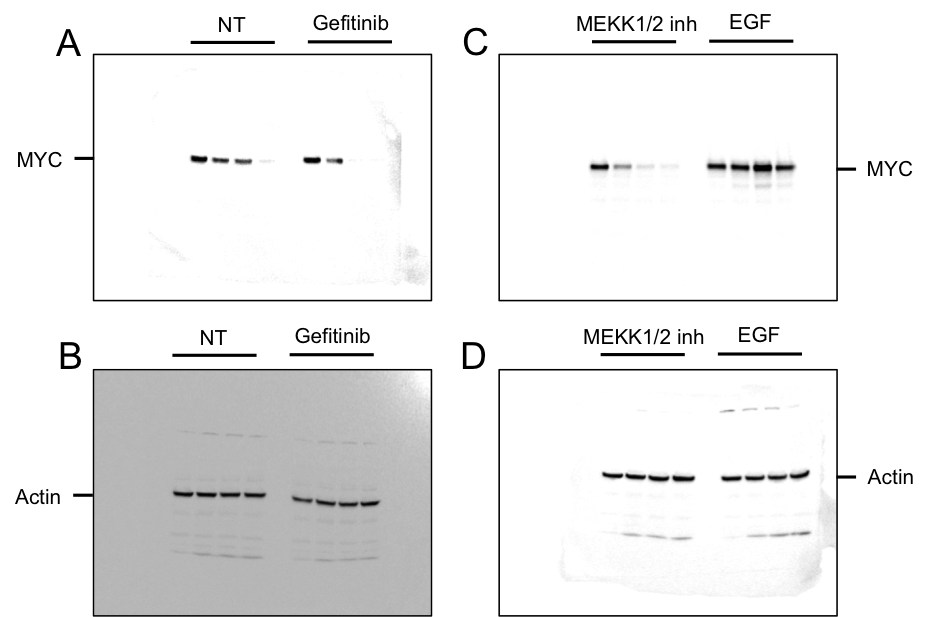
**
